# Occupational heat stress and associated productivity loss estimation using the PHS model (ISO 7933): a case study from workplaces in Chennai, India

**DOI:** 10.3402/gha.v7.25283

**Published:** 2014-11-04

**Authors:** Karin Lundgren, Kalev Kuklane, Vidhya Venugopal

**Affiliations:** 1 Thermal Environment Laboratory, Division of Ergonomics and Aerosol Technology, Department of Design Sciences, Lund University, Lund, Sweden; 2 Department of Environmental Health Engineering, Sri Ramachandra University, Chennai, India

**Keywords:** occupational heat stress, productivity, international standards, India, climate change

## Abstract

**Background:**

Heat stress is a major occupational problem in India that can cause adverse health effects and reduce work productivity. This paper explores this problem and its impacts in selected workplaces, including industrial, service, and agricultural sectors in Chennai, India.

**Design:**

Quantitative measurements of heat stress, workload estimations, and clothing testing, and qualitative information on health impacts, productivity loss, etc., were collected. Heat strain and associated impacts on labour productivity between the seasons were assessed using the International Standard ISO 7933:2004, which applies the Predicted Heat Strain (PHS) model.

**Results and conclusions:**

All workplaces surveyed had very high heat exposure in the hot season (Wet Bulb Globe Temperature $\bar{x}$ =29.7), often reaching the international standard safe work values (ISO 7243:1989). Most workers had moderate to high workloads (170–220 W/m^2^), with some exposed to direct sun. Clothing was found to be problematic, with high insulation values in relation to the heat exposure. Females were found to be more vulnerable because of the extra insulation added from wearing a protective shirt on top of traditional clothing (0.96 clo) while working. When analysing heat strain – in terms of core temperature and dehydration – and associated productivity loss in the PHS model, the parameters showed significant impacts that affected productivity in all workplaces, apart from the laundry facility, especially during the hot season. For example, in the canteen, the core temperature limit of 38°C predicted by the model was reached in only 64 min for women. With the expected increases in temperature due to climate change, additional preventive actions have to be implemented to prevent further productivity losses and adverse health impacts. Overall, this study presented insight into using a thermo-physiological model to estimate productivity loss due to heat exposure in workplaces. This is the first time the PHS model has been used for this purpose. An exploratory approach was taken for further development of the model.

When the human core temperature reaches or exceeds 38°C, there are well-documented physiological effects on the human body, posing risks to some organ systems and also making it progressively harder to work productively, especially physically (1). A natural reaction of a working person to heat is to reduce physical activity, which reduces the body’s internal heat production (2–5) . An outcome of this natural preventive mechanism is reduced work capacity, which results in lower productivity (2). Occupational health is affected by climate change by increasing temperatures, among other impacts (6, 7). The Intergovernmental Panel on Climate Change in its latest report states that hot days, hot nights, and heat waves have become and will become more frequent over most land areas (8). In addition, it has been observed that the increasing temperature of ocean surface water will create more evaporation of water resulting in higher absolute humidity of the atmosphere over most land areas (6). High humidity affects human thermal regulation because of reduced evaporation of sweat.

Heat exposure has been shown to affect outdoor workers in India (9, 10) and associations between high temperatures and mortality have been established (11, 12). Dunne et al. estimated that environmental heat stress has already reduced the global labour capacity significantly in peak months with a further predicted reduction of 80% by 2050 (8). Without adaptation, the economic losses of reduced labour productivity relative to baseline could be significant (3) and become the most costly impact of climate change (13).

This paper explores the problem of heat stress in selected workplaces, including industrial, service, and agricultural sectors in Chennai, India. During the hottest month in Chennai, temperatures are already high enough to cause major loss of hourly work capacity and this situation will worsen for many jobs in the face of future climate change. Data were collected in the cooler and hotter seasons to estimate the current occupational heat stress situation and associated productivity impacts between the seasons. The study was conducted using the heat balance equation and the Wet Bulb Globe Temperature (WBGT, ISO 7243) index (14). Heat strain and associated reduced labour productivity between the seasons was assessed using the International Standard ISO 7933:2004, which adopts the Predicted Heat Strain (PHS) model (15). This physiological model has been used in previous studies, for example, for developing heat surveillance systems (16, 17).

## Background

### Climate profile of Chennai

Chennai, the capital city of Tamil Nadu, is located near the equator and experiences a hot and humid climate throughout the year. Temperature range in the hot months of May and June is between 35 and 40°C and the month of May records the highest temperatures reaching 45°C causing potential risks to local workers of developing heat-related disorders. Winter occurs from November to February, with January being the coolest month of the year (15–22°C). During the monsoons, from June to September, Chennai receives abundant rainfall and humidity is high with a relative humidity of between 80 and 96% (18). Based on this, the field work was carried out during the periods January–February and April–May to assess productivity loss between the hot and cool seasons.

### Description of workplaces

#### Cookie factory

**Fig. 1:**
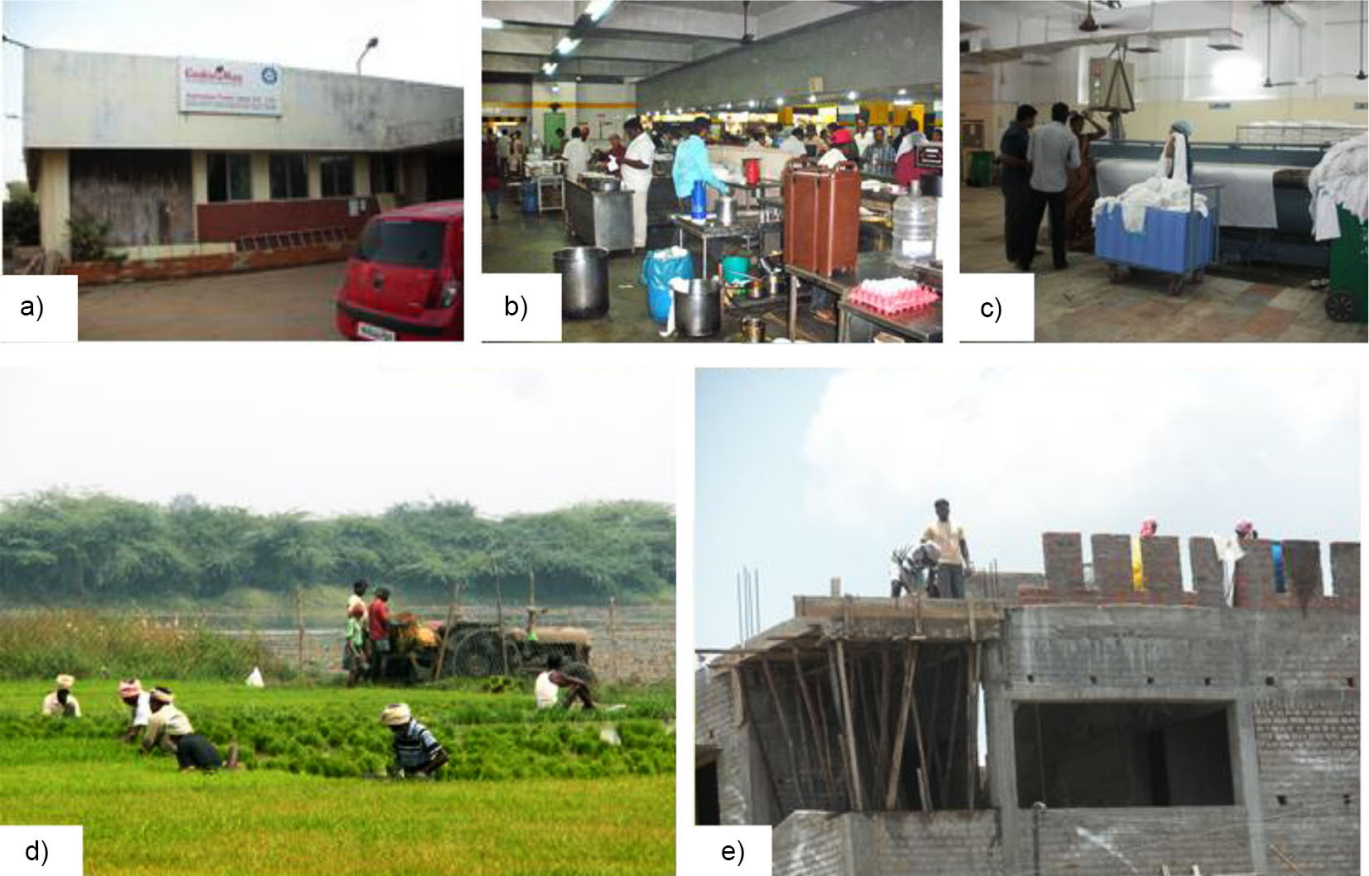
Pictures of workplaces: a) outside view of cookie factory, b) food serving area of the canteen, c) drying machines at the laundry, d) agricultural field, e) roof of construction building.

There are different sections and work tasks in the production line. The first section is the raw material receiving and storage area, involving such tasks as lifting of boxes, carrying, and bending. The tasks are performed in semi-outdoor conditions. In the next section, the pre-scaling area, different raw materials are received and weighed and sent to the mixing area. Mixing is done both manually and mechanically. The cookies are baked in the production area. The finished products are received in the packing area and work is performed in standing positions. Finally, in the dispatch area, the packed goods are stored and materials are loaded manually into vehicles (Fig. 1).

#### Canteen

The canteen has different sections that prepare western and south Indian food. In the raw material receiving and storage area, vegetables and food items are received and stored and a clerk maintains the records. In the vegetable cutting area, vegetables are cleaned and cut. In the food preparation area, breakfast items and rice, vegetables, and other items are cooked. Tea and coffee are served throughout the day. In the rice preparation area, rice is cleaned and fed into boilers. In the food serving area, plates are collected and cleaned manually by cleaning workers. Finally, in the billing section, computer billing is carried out at different counters that are manned by clerks who are standing (Fig. 1).

#### Laundry facility

The laundry facility serves students, faculty, and the hospital. The laundry from the hospital is decontaminated before washing and hot pressing. In the hot press section, large sheets are fed into the press and the work is performed manually. In the washing section, clothes are fed into the machine and unloaded manually. After washing, the clothes are loaded onto a trolley and taken to the drying section. After drying, the clothes are manually ironed and folded (Fig. 1).

#### Agriculture

The agricultural area is located in a village outside Chennai where crops are cultivated according to the seasons. There are different phases in cultivation: preparation of land for cultivation, sowing, watering, clearing weeds, pest control, fertilization, crop maintenance, and harvesting. The work usually starts in the early morning and is completed before noon. In preparing the land, the workers perform intense shovelling, although sometimes tractors are used. Manual work is performed in the other phases. Workers spend most of their time bending during planting and harvesting (Fig. 1).

#### Construction

There are different work tasks involved in construction, such as intense shovelling, carrying and disposal of debris, and cutting of iron bars. There are different categories of workers involved: manual labourers, masons, stone cutters, bar bending workers, painters, electricians, etc. The amount of time spent exposed to the hot sun varies depending upon the nature of work performed. Most of the workers are migrants from poorer Indian states. Besides working in the construction site, the workers are also constantly exposed to heat in their temporary housing. The houses are made of metal sheets that do not provide relief from heat exposure after work (Fig. 1).

## Methods

Six parameters were measured in this study to determine the body heat balance. Four were environmental parameters: air temperature, radiant temperature, humidity, and air movement. Two were non-climatic parameters consisting of clothing and metabolic rate (4).

### Environmental parameters and the WBGT index

The WBGT (ISO 7243) is a direct index simulating the response of the human body to heat stress and is widely used in the assessment of occupational heat stress (4, 14, 19). The international standard for WBGT uses a formula based on measurements of three temperature variables: *T*_a_, the air temperature measured with a shielded thermometer; *T*_g_, the globe temperature which is the temperature inside a black globe that is strongly affected by heat radiation; and *T*_nw_, the natural wet bulb temperature, which is measured in the actual sun and wind exposure situation, with a wet cloth over the bulb representing the impact of evaporation (14). In this study, *T*_a_, *T*_nw_, *T*_g_ and relative humidity (RH) were collected using the 3 M™ QUESTemp°™ 32 heat stress meter (accuracy of *T*_a_, *T*_nw_ and *T*_g_:±0.5°C; RH:±5%) together with personal exposure measurements using LASCAR dataloggers (EL-USB-2-LCD^+^, accuracy of *T*_a_: ±0.3°C; RH: ±2%).

### Clothing

The work clothing was tested for insulation and evaporative resistance on thermal manikins at Lund University, Sweden; Loughborough University, UK; and Hong Kong Polytechnic University as part of another research project (20). Five sets of work clothing from Chennai were included in the project and in this analysis based on their widespread use in many workplaces.

### Metabolic heat production

Work activity was calculated according to heart rate conversion to metabolic rate (21) if possible. Heart rate was measured using POLAR pulse monitors (Käyttö 4000 RUD 11.93 STK model, with readings every 15 sec). Heart rate recordings are affected by factors such as physical fitness, environmental temperature, nutrition, health status, psychological factors, and size of active muscle mass; all of which add complexity (22). In addition, the methods of estimating the metabolic rate involve many uncertainties because the worker usually performs a combination of activities and hence, the metabolic rate fluctuates during the working day (23). Because of these aspects, and because of the limited measured data (not available for agriculture and construction), observations using the ISO 7243 reference table complemented the measured data (14).

### Heat strain and labour productivity

Previous papers examining climate change, heat, and work productivity have used productivity measures such as the collection of ‘bundles’ (24), gross domestic product (GDP) together with climate data (8) and productivity as a function of the ISO 7243 standard guidelines (3, 8, 25) or the thermal comfort standard, the Predicted Mean Vote Index (26). In addition, the time taken to achieve production targets, company records, overtime, and the use of interviews and questionnaires about workers’ perceptions of heat and work productivity have been applied (27). Finally, thermal chamber studies have been conducted (28).

The PHS (ISO 7933) (15) is a rational physiological model based on data on physiological responses of subjects in hot conditions from laboratory studies. The model makes it possible to predict sweat rate and core temperature and calculate duration exposure limits (29–31) . In this study the heat strain prediction was utilized for the thermo-physiological responses’ associated productivity losses. Productivity in this paper is based on the following outputs in the PHS model: time to reach a core temperature of 38°C and maximum water loss that will indirectly affect productivity. The time to reach a core temperature of 38°C for an average worker and dehydration limits are seen as critical parameters in the model. Physiological parameters have previously been found to be indicators of productivity loss. For example, Nag et al. found that when the core temperature reaches 38°C, if the worker is able to self-pace, s/he will adjust the pace and slow down (5). This translates into an automatic productivity loss. Internationally, the WHO 1969 guideline on the allowable limit core temperature of 38°C was developed to protect most workers (32). Dehydration affects productivity by elevating core temperature with an increase of about 0.1–0.2 degrees per percentage of dehydration (33). Dehydration also causes cardiovascular strain, resulting in elevated heart rates. Dehydration deficits of 2% of body mass can adversely impact aerobic performance, orthostatic tolerance, and cognitive function (33) and the limit used for dehydration is set at 3% of body mass for industrial workers (34). Moreover, Wasterlund et al. found that every percentage water loss in forest workers resulted in a 12% reduction in productivity (35).

In all workplaces in the PHS simulation, it was estimated that the worker was able to take three 15-min breaks (one in the morning and two in the afternoon) and one 50–75-min lunch break in between during 8 hours. Resting means the metabolic rate was set at 100 W/m^2^. Environmental data input for resting was considered the coolest room or in the shade, as no workplace had air conditioned resting areas. An air velocity of 1 m/s, simulating a slow walk, was used in the model as body movement creates air flow over the body (19); and an air velocity of 0.3 m/s was used when resting. All workers were considered acclimatized with free access to water (however, in agriculture, the workers usually carry their personal water bottle of about 1.5 litres out to the field, consequently making them more vulnerable to risks of heat stress).

## Results

The average basic environmental parameters and standard deviations for each workplace in the cooler season (January–February) and hot season (April–May) can be seen in Table 1. The results are averages of measurements in the different sections at each workplace.

**Table 1:** Environmental parameters and WBGT index measurements

Parameter	Cookie factory		Canteen		Laundry facility		Agriculture		Construction	

	Cooler	Hot	Cooler	Hot	Cooler	Hot	Cooler	Hot	Cooler	Hot
*T*_a_	30.2 (s.d. 1.3)	35.6 (s.d. 2.4)	33.7 (s.d. 2.3)	36.8 (s.d. 1.7)	30 (s.d. 0.5)	32.7 (s.d. 0.3)	28.9 (s.d. 0.5)	32.0 (s.d. 0.3)	30 (s.d. 0.0)	34.2 (s.d. 0.8)
*T*_g_	30.5 (s.d. 4.6)	36.0 (s.d. 4.1)	35.2 (s.d. 3.6)	38.1 (s.d. 3.0)	30 (s.d. 1.4)	32.7 (s.d. 0.2)	38.5 (s.d. 2.1)	39.5 (s.d. 0.6)	32 (s.d. 1.3)	37.6 (s.d. 5.4)
*T*_nw_	22.8 (s.d. 0.9)	27.4 (s.d. 1.5)	27.1 (s.d. 1.3)	30.0 (s.d. 0.9)	21.2 (s.d. 0.3)	26.5 (s.d. 0.8)	24.4 (s.d. 0.3)	26.5 (s.d. 0.4)	23.5 (s.d. 0.4)	25.3 (s.d. 0.7)
RH	54% (s.d. 3.6)	56.4% (s.d. 3.8)	59.1% (s.d. 6.5)	55.6% (s.d. 1.1)	62.3% (s.d. 3.8)	54.3% (s.d. 7.4)	58.5% (s.d. 3.9)	50.8% (s.d. 1.9)	53.6% (s.d. 2.0)	44.4% (s.d. 2.6)
**WBGT**	**25.0 (s.d. 1.9)**	**30.0 (s.d. 1.7)**	**29.5 (s.d. 2)**	**32.0 (s.d. 2.3)**	**24.3 (s.d. 0.3)**	**28.0 (s.d. 0.6)**	**28.0 (s.d. 0.6)**	**29.6 (s.d. 0.4)**	**25.6 (s.d. 0.3)**	**28.7 (s.d. 1.5)**

Climate Index.

A two-tailed paired students *t*-test proved a statistically significant difference in WBGT values between the cooler and hotter months (*p*=0.002566). Though the productivity loss associated with following ISO 7243 guidelines (estimate value from linear regression model *ß*=0.5924), the increased WBGT values are not statistically significant (*p*=0.2541), which could be partly due to the small sample size.

### Clothing

The mean insulation and standard deviation results from standing measurements from the three laboratories are presented in Table 2.

**Table 2:** Clothing description and measured insulation and evaporative resistance (20)

Ensemble	Workplaces	Permeability index (*i*_m_) (mean of 3 measurements)	Clothing basic insulation (*I*_cl_) clo (mean and s.d. of 3 laboratories)
Churidar (f)	Laundry and cookie factory	0.38	0.58 (s.d. 6.23%)
Churidar+shirt and towel on head (f)	Construction, agriculture, and canteen	0.34	0.74 (s.d. 4.86%)
Saree (f)	Laundry and cookie factory	0.34	0.74 (s.d. 7.28%)
Saree+shirt and towel on head (f)	Construction, agriculture, and canteen	0.31	0.96 (s.d. 7.80%)
Shirt and trousers, with towel on head (m)	Agriculture and construction	0.33	0.61 (s.d. 2.53%)

f=female, m=male.

Most clothing was made of cotton and had high insulation values in relation to the heat exposures. Female clothing with shirt and head cover had higher insulation values compared to male workwear. In Chennai, it is common for female workers to wear traditional clothing – churidars and/or sarees – with a protective shirt on top and head cover while working in some workplaces, which decrease ventilation. When this was the case, the female workwear had considerably higher insulation and evaporative resistance than male workwear (20).

### Metabolic heat production

The mean heart rate results and standard deviations, together with observations using the ISO 7243 reference table are presented in Table 3.

**Table 3:** Estimated average metabolic rates from heart rate data and observations at various workplaces in Chennai, India

Site, total numbers of observed workers (*N*) and gender	Work tasks involved (from observations)	Profession and average heart rate (b/min) measured	Average metabolic rate calculated (ISO 9886, 2004b) from heart rate (W/m^2^)	Average metabolic rate (W/m^2^) from ISO 7243 and metabolic class
Industrial: Cookie factory (*N*=8; m=7 f=1)	Sustained hand and arm work, pushing/pulling/lifting light weight boxes, bending, mixing, walking speed 2.4–5.5 km/h.	Mixer: 97 (s.d. 6.1) Packer: 91 (s.d. 5.7) Packer: 95 (s.d. 9.1) Baker: 115 (s.d. 13.6)	178 144 169 260	**188** (130<*M*<200) **Moderate**
Service: Canteen (*N*=9; m=9)	Sustained hand and arm work, standing cooking, preparation, lifting/pushing/pulling light weight boxes, bending, walking speed 2.4–5.5 km/h.	Cook: 97 (s.d. 11.3)	170	**170** (130<*M*<200) **Moderate**
Service: Laundry (*N*=9; m=2 f=7)	Sustained hand and arm work, manual loading/unloading, ironing, folding and packing walking speed 2.4–5.5 km/h.	Dryer: 118 (s.d. 13) Washer: 90 (s.d. 17.5) Washer: 100 (s.d. 6)	245 120 177	**181** (130<*M*<200) **Moderate**
Agriculture (*N*=4; m=1 f=3)	Preparation of land for cultivation, sowing, watering, weeding, pest control, fertilization, crop maintenance and harvesting, bending, walking speed 2.4–5.5 km/h.	–	–	**190** (130<*M*<200) **Moderate**
Construction (*N*=16; m=16)	Intense arm and trunk work: shovelling, carrying and disposal of debris, cutting of iron bars, pushing and pulling heavy carts, walking speed 5.5–7 km/h.	–	–	**220** (200<*M*<260) **Heavy**

f=female, m=male.

Average metabolic rate for each workplace.

The heart rate subjects in Table 3 were only men because of cultural limitations. Most were under the age of 30; one was 52, and one was 60. Measurement time varied between 3 and 5 hours. Because of this limited data, observations using the ISO 7243 reference table complemented the heart rate data. The very high results from the heart rate data – 260 and 245 W/m^2^ – were mainly due to very high heat exposure (33): the baker in the cookie factory and the dryer in the laundry, respectively.

### Anthropometric data

The data consisted of average values measured on selected workers at the workplaces (66 males and 11 females). The average male was 167 cm (SD 7.4) and 64 kg (SD 12.6), and the average female, 150 cm (SD 4.1) and 56 kg (SD 3.9) was used as data input into the PHS model.

### Heat strain and labour productivity using the PHS model (ISO 7933)

**Fig. 2:**
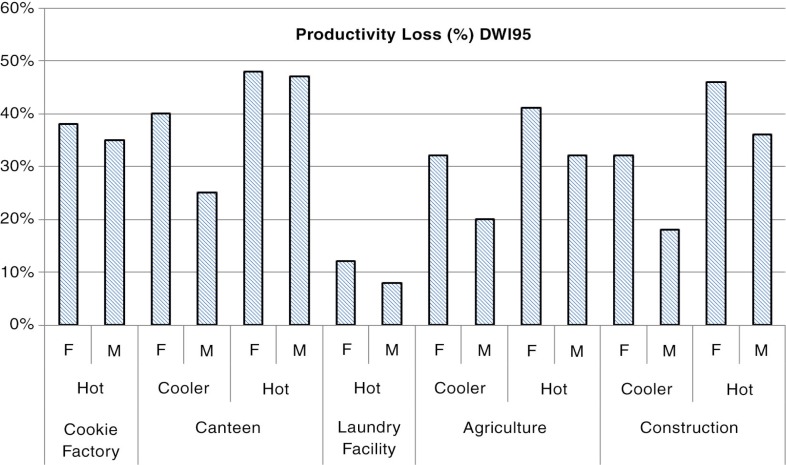
Results from PHS simulations: predicted productivity loss estimated from Dwl95 (the maximum water loss to protect 95% of the working population). F=female, M=male.

Figure 2 illustrates the output from the PHS model, which predicts the time to reach maximum water loss for the average healthy working person. The worker should rest and drink when this limit is reached, affecting productivity. The sweat rates are very high in the hot season, especially in the canteen where workers should rest half the workday to stay healthy. If water is not readily available this will translate into risks for heat disorders (4).

**Fig. 3:**
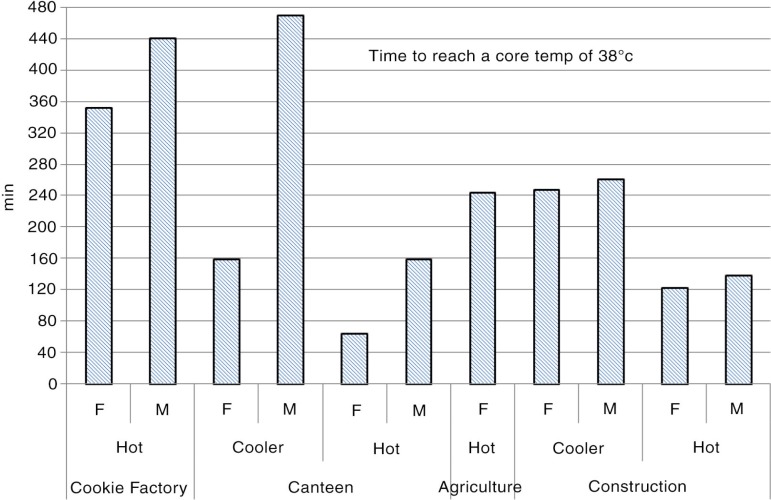
Results from PHS simulation: the predicted time to reach a core temperature of 38°C. F=female, M=male.

In Fig. 3, the predicted time in minutes (total 8 hours) to reach a core temperature of 38°C is shown. As mentioned previously, when the core temperature reaches 38°C, an automatic productivity decline has been observed in India if the worker is able to self-pace.

### Limitations of the study

There are no comprehensive physiological models to estimate productivity loss in hot environments and therefore, there is a need to revise the PHS model. Validation studies are required on the relationship between PHS predictions and productivity. Although, one has to keep in mind when using a thermo-physiological model that it does not account for other aspects of the work environment that affect productivity, such as light and noise (36, 37), cognitive function (33), the psycho-social environment, long commutes, lack of sleep, and stress. In addition, the PHS model also assumes an initial core temperature of 36.6°C. However, most of the workers had travelled to work or lived in hot dwellings and had therefore been exposed to heat beforehand. As a result, performance may be affected before the indicators in the model.

In general, practical considerations had a strong influence on the field research. For example, the opportunity sampling conducted with the heart rate and anthropometric data does bias how representative the sample is of the wider population. Also, the study provides only a snapshot of the reality for these workers as each field visit lasted between 4 and 6 hours during midday and not for a full working day or over several weeks.

## Discussion

All workplaces surveyed had very high heat exposure in the hotter months, often reaching WBGT values over the international standard limit values (a WBGT of around 27) (14) where, depending on the metabolic rate, acclimatization state, and clothing; actions should be taken. Most workers had moderate to high workloads, some in direct sun exposure. All workplaces studied, apart from the laundry facility, reached critical thermal conditions in the PHS model, especially in the hot season, affecting productivity. In the canteen in the hot season, the limit core temperature was reached in only 64 min for women, whereas men could work for another 95 min before reaching this limit. As mentioned previously, this is mainly due to the different clothing practices. This health impact can be significantly reduced by allowing workers to self-pace their work; providing safe drinking water and regular breaks throughout the day; and awareness of heat stress symptoms. The real danger is when the work is externally paced (e.g. by machinery, quotas, peer pressure), as workers will push themselves beyond the safe limit and become at risk of developing heat disorders. At most risk are workers who are poorly hydrated, unacclimatized, or physically unfit.

Clothing was found to be problematic, with high insulation values in relation to the heat exposure (although within the PHS validity range) (29). A sex difference in heat exposure was observed in the population studied because females were more vulnerable due to the traditional clothing worn under protective layers in the construction, agriculture, and the canteen workplaces. Female workers’ clothing practices trap a saree’s layers beneath tighter textile decreasing air, vapour permeability and ventilation, and increasing the clothing’s insulation, which generates a higher heat load for these women. On the other hand, the ventilation effect of the traditional clothing such as the saree is likely underestimated as the PHS model is based on Western clothing data. Overall, it is a local cultural practice that is hard to change and requires more research.

### Preventive approaches

In the questionnaire, most workers reported health problems due to heat exposure, including thirst, heavy sweating, muscle cramps, tiredness/weakness, dizziness, and headaches, and a few reported problems with nausea/vomiting and fainting. Problems with meeting production targets in the hotter months were usually compensated for by working overtime. Locally in workplaces, there were numerous technical, behavioural, and managerial methods to reduce heat exposure. Apart from self-pacing and rest, traditional methods, including mainly drinks and diet, dominated the coping mechanisms. These were mainly drinking buttermilk (fermented yoghurt drink) followed by fruit juice, coconut water, and fermented rice. Coconut water is a drink seen in the literature as a potential natural alternative to artificial sports drinks due to its richness in potassium, sodium, chloride, and carbohydrates (38, 39).

Other methods included ventilation, exhaust fans, and shade structures. Sustainable, low-cost and adaptable cooling methods must be further studied and adopted together with education and awareness of heat stress.

### Climate change dimensions

India is already experiencing a warming climate, and climate change risks are high and multidimensional. Warming trends and increasing temperature extremes have been observed across most of the South Asian region. The average temperature has been increasing at a rate of 0.14–0.20°C per decade since the 1960s, combined with a rising number of hot days and warm nights (40). There is a risk of an increase in mean, minimum, and maximum temperatures of 2–4°C (41). Adding these temperature projections to the current conditions at the workplaces studied, will have profound implications for the workers’ health and productivity, in particular in the outdoors where cooling options are limited. Today, the thermal stress at the workplaces is already at the borderline of human tolerance and may not need to increase much to result in a drastic drop in productivity (8, 42).

## Conclusion

All workplaces surveyed had very high heat exposure in the hotter months, often reaching WBGT values above the international standard limit values (measured WBGT $\bar{x}$=29.7) (ISO 7243:1989) for working safely. Most workers had moderate to high workloads (170–220 W/m^2^), some in direct sun exposure. Clothing was found to be problematic, with high insulation values in relation to the heat exposure. Females were more vulnerable due to work clothing practices. When analysing heat strain and associated productivity loss in the PHS model apart from the laundry facility, the parameters showed significant impact in all workplaces, especially during the hot season, affecting productivity. For example, in the canteen in the hot season, the predicted limit core temperature was reached in only 64 min for women. If self-pacing is possible and water widely available this impact can be significantly reduced. Nevertheless, with expected climate change, additional preventive actions have to be taken in these workplaces immediately to mitigate further productivity losses. Overall, this study is presented as an exploratory study into using a thermo-physiological model as the basis to estimate productivity loss due to heat exposure in workplaces. The PHS model is not designed for this, although previous studies have linked heat strain parameters with productivity loss.
